# Spectral Characteristics of Dissolved Organic Matter in Farmland Soils around Urumqi, China

**DOI:** 10.3390/toxics11040376

**Published:** 2023-04-16

**Authors:** Jianhua Zhu, Jia Duo, Zizhao Zhang, Liang Pei, Wenfeng Li, Rehemanjiang Wufuer

**Affiliations:** 1 Geological Environment Monitoring Institute of Xinjiang Uygur Autonomous Region, Urumqi 830091, China; 2 Xinjiang Key Laboratory of Environmental Pollution and Bioremediation, Xinjiang Institute of Ecology and Geography, Chinese Academy of Sciences, Urumqi 830011, China; 3 National Engineering Technology Research Center for Desert-Oasis Ecological Construction, Xinjiang Institute of Ecology and Geography, Chinese Academy of Sciences, Urumqi 830011, China; 4 School of Geology and Mining Engineering, Xinjiang University, Urumqi 830046, China

**Keywords:** farmland soil, fluorescence characteristics, fulvic acid, humic acid

## Abstract

The dissolved organic matter (DOM) is one of the most sensitive indicators of changes in the soil environment, and it is the most mobile and active soil component that serves as an easily available source of nutrients and energy for microbes and other living organisms. In this paper, DOM structural characteristics and main properties were investigated by three-dimensional fluorescence spectroscopy (EEM) and UV–visible spectrum technology in the farmland soils around Urumqi of China, and its possible sources and pathways were analyzed by spectroscopic indices. The results showed that humic-like substances were the main composition of the soil DOM, and its autogenesis characteristics were not obvious. Main DOM properties such as aromatability, hydrophobicity, molecular weight, molecular size, and humification degree in the southern region of Urumqi were higher than those of the northern region of Urumqi and Fukang in China, and higher on the upper layers of the soil (0–0.1 and 0.2 m) than in the deeper layer (0.2–0.3 m).This may be because the tilled layer is more subjected to fertilization and conducive to microbial activities. The spectroscopic analysis showed that the source of DOM of these regions is mainly from microbial metabolites. These results provide basic scientific data for the further research on the environmental chemical behavior of pollutants and pollution control in this region.

## 1. Introduction

Dissolved organic matter (DOM) is composed of humic acid, fulvic acid, and other organic materials which have abundant biological elements such as nitrogen, carbon, and phosphorus, and which exist extensively in various water, soil, and sediment environments [1,2]. It is closely related to the microbial metabolic process and the biological, chemical, and physical properties of the soil [3]. Soil DOM constitutes a rather small part of the soil organic matter, but it is the most mobile and active soil component, which can indicate the changes in soil processes and serve as a nutrient and energy source for microorganisms [3,4,5,6]. More importantly, DOM has influence on the soil negative charge denitrification process and acid-based reactions in the soil solution, and plays an important role in the biogeochemical carbon cycle [3]. Soil DOM has a wide range of sources [7,8], such as plant and animal residues, plant secretions, and industrial and agricultural organic wastewater [9,10,11].

Different DOM components in soil play an important role in agricultural production. DOM contains organic molecular mixtures with different structures and molecular weights, except nutrients such as C, N and P, for the growth of crops [9,12]. They contain many kinds of active functional groups, which affect soil fertility and microbial activities through different pathways [13]. Humic acid in soil DOM can greatly enhance the respiration of plants, improve the permeability of cell membranes of plants, and facilitate the absorption of nutrients by plants [14]. The organic colloid in soil DOM with a large number of negative charges has strong adsorption capacity [15]. Consequently, the cation exchange and water absorption capacity can be increased by several times or even tens of times in the clay particles by adsorbing a large number of cations and water [16,17]. These indicate that some components in soil DOM can play a significant role in improving soil fertility and buffering the soil acid–base balance. The humus in soil DOM has strong complexation ability and combines with environmental pollutants, such as potentially toxic elements, polycyclic aromatic hydrocarbons, pesticides, polychlorinated biphenyls, and herbicides, which affects their migration, toxicity, and bioavailability [18,19,20]. Therefore, understanding chemical characteristics of DOM in farmland soil could help us to better understand the transfer of major elements, nutrients, and water within farmland environments.DOM of autochthonous and allochthonous origins differ in properties and biogeochemical behavior. Allochthonous DOM may drive shifts in microbial community composition, whereas autochthonous DOM seems to affect the microbial community composition only transiently [21,22]. Therefore, the source of DOM should be given more attention in the farmland. The unique climate features, as well as low levels of water and nutrients, make the semi-arid regions prone to desertification [23]. To reveal the composition and source of farmland soil DOM will be particularly important in the arid regions of Northwest China due to the challenges of water deficiency, low water utilization rate, and low fertility in soil. DOM information is of particular importance for climate-sensitive soil ecosystems such as arid regions.

Joint use of multiple techniques to measure DOM properties is necessary as no single technique is capable of fully characterizing DOMs [24,25]. At present, spectrum, chromatography, energy spectrum, mass spectrum, and other characterization technologies have been gradually developed to characterize DOM properties and components [26,27,28]. Among them, spectral technology, due to its simple operation, is widely used to obtain DOM information such as aromatability, molecular weight, humification index, fluorescence components, and molecular spatial configuration [29,30,31]. In this study, three-dimensional fluorescence spectroscopy coupled with UV–visible spectrum technology were used to characterize the structural composition and important properties such as hydrophobicity, aromatability, molecular weight, and polarity of the farmland soil DOM around Urumqi city, and its sources and pathways were analyzed by spectroscopic indices.

Over the past several decades, the content, structure, and source of soil DOM have been widely investigated [9,10,12,32]. The majority of those studies have focused on forest soils, wetlands, sediments, and water [33,34]. Less attention has been paid to farmland soil DOM, particularly in arid and semi-arid regions. Serious soil erosion and salinization have made the scarcity of the limited arable land resources increasingly prominent in these regions. Therefore, further studies are still necessary to explore DOM composition and sources in farmland of arid and semi-arid regions. We need to better understand how DOM components vary and which site factors exert the greatest influence on DOM dynamics in farmland of this region, given the importance of DOM to biogeochemical cycles of farmland ecosystems. In this study, the important structural properties, its sources, and pathways of the farmland soil DOM around Urumqi of China were analyzed by spectroscopic indices in order to provide a scientific basis for understanding the migration and transformation processes of potentially toxic pollutants in the farmland of arid and semi-arid regions. More importantly, the researching results can provide important geochemical evidence for rational agricultural programming, planting, soil improvement, and scientific fertilization in the arid regions of Northwest China.

## 2. Materials and Methods

### 2.1. Site Description

**Figure 1:**
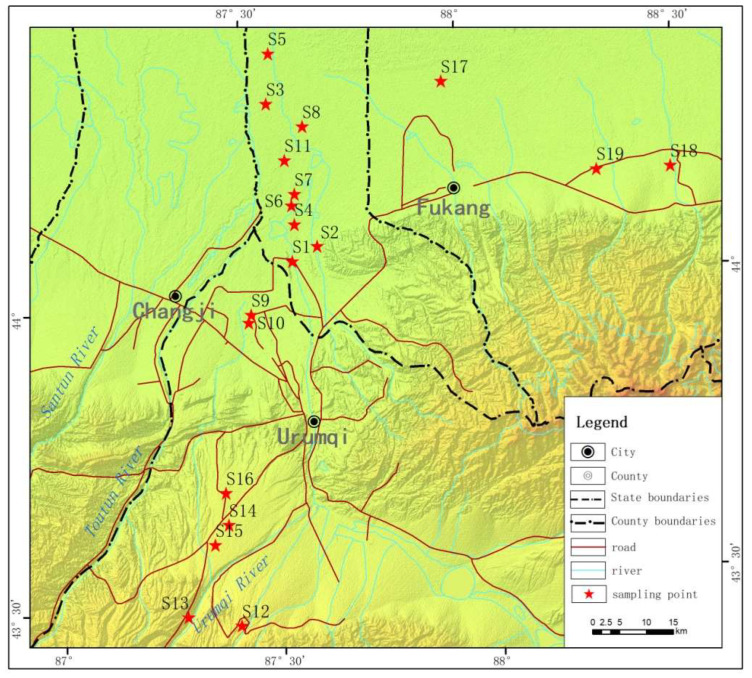
The Map of the sampling points.

Urumqi, the capital of the Xinjiang Uygur Autonomous Region, China, is located in the arid region of Northwest China and the center of the Eurasian continent, with an average altitude of 800 m. The geographic coordinates of the city are 86°37′33″–88°58′24″ E, 42°45′32″–44°08′00″ N [35]. The city belongs to the semi-arid temperate continental climate with an annual average precipitation of 2100 mm and an annual mean temperature of 7.3 °C. The topography is complex, and the terrain slopes downward from the south to the north and extends along the rivers in a zonal pattern. According to the third national land survey in 2019, Urumqi has 71,382.8 hectares of farmland. The area of high-quality farmland is about 46,960 hectares and is concentrated on the alluvial plain along Urumqi River and Toutun River. They are mainly distributed in Liushihu and Qinggeda lake townships in Xinshi District, Yangmaogong town and Changshanzi town in Midong district, Donggou township and Xigou township in Dabancheng District, Yongfeng township, Banfanggou township, and Shuixigou town in Urumqi County. Our sample collection is distributed in these districts, as shown in Figure 1 and Table 1.

### 2.2. Site Investigation and Sample Collection

The sampling was conducted in autumn, 2019.Three study areas around Urumqi city (northern Urumqi, southern Urumqi, and Fukang) were chosen with different soil properties and crops types. As shown in Figure 1, soil samples of 19 crop types were collected and marked 1S (Site 1)–19S (Site 19), respectively. The crops in the northern Urumqi region are mainly sweet potatoes, corn, cotton, and greenhouse vegetables. The crops in the southern Urumqi region are mainly potatoes, onion, flowers, and greenhouse vegetables. The crops in Fukang are mainly cotton and corn. Soil samples were collected with a 7.5-cm-diameter auger at each sampling site. We randomly collected 5 samples with an S-shaped pattern in each farmland and mixed them into a single sample. A total of 57 soil samples were collected from 19 agricultural land sites at different layers (0–0.1, 0.1–0.2, and 0.2–0.3 cm). All soil samples were immediately transported to the laboratory, and visible residues and roots were removed manually before passing them through a 2 mm sieve. Soil samples were then stored in a refrigerator (<4 °C) to measure the characteristics of the dissolved organic matter (DOM) in the soil.

### 2.3. DOM Extraction in Soil

The DOM extraction of soil samples was carried out by the soil and water vibration method. The collected soil samples were dried, grounded, and screened through 80 mesh for later use. A mass of 3.00 g of the treated soil sample was accurately weighed into a 250 mL conical flask, followed by the addition of 30 mL of ultra-pure water to adjust the ratio of soil to water to 1:10. The samples were placed in a constant temperature shaker for 18 h (25 °C, 200 r·min^−1^) and transferred to 50mL centrifuge tubes. After being centrifugated at 5000 r·min^−1^ for 10 min, the supernatant was taken and filtered through a 0.45 μm filter membrane to obtain the DOM solution of the soil samples, and was stored in a refrigerator at 4 °C for further analysis within 7 days [36].

### 2.4. Spectroscopic Analyses

F-7000 molecular fluorescence spectrometer (Hitachi, Japan) was used to detect the fluorescence characteristics of the DOM of the soil samples. The scanning speed was 1200 nm·min^−1^, and the scanning spectrum instrument was automatically corrected. The fluorescence spectrophotometer scanned over an excitation range of 200–500 nm at 5 nm intervals and an emission range of 200–550 nm at 5 nm intervals. The temperature of the reaction system was 22 °C, and the deionized water blank was subtracted from the measured results. A Shimadzu UV-3600 photometer was used to determine the UV–visible spectrum of the DOM, scanning over an excitation range of 200–500 nm at 1 nm intervals. Ultrapure water was used as the blank. All tests were repeated three times, and typical spectra are shown from one of the triplicate examinations.

### 2.5. Spectroscopic Indices and Data Analysis

General features and three optical indices were used to further describe the compositional characteristics of soil DOM based on the corrected absorbance and EEM fluorescence data. SUVA_254_, SUVA_260_, and SUVA_280_ are the absorption coefficients of unit DOC concentrations at wavelength 254 nm, 260 nm, and 280 nm, and they are used to characterize the aromaticity, hydrophobicity, and molecular weight of DOM molecules, respectively [37,38]. A_250_/A_365_ is the absorbance ratio at 250 and 365 nm and is used to estimate the size of DOM molecules. A_300_/A_400_ is the absorbance ratio at wavelengths 300 and 400 nm and is used to characterize the humification degree of DOM molecules [39]. Fluorescence index (FI), the ratio of the emission intensity at 450 nm–500 nm, is used to reflect the relative microbial (>1.9) or terrestrial plant contribution (<1.4) [40]. Biological index (BIX) is an indicator of the relative contribution of the recently microbially produced DOM, calculated as the ratio of emission intensity at 380–430 nm [41].

Averages and standard deviations were determined using Excel 2020 (Microsoft Office 2020; Microsoft, Redmond, WA, USA). The figures were drawn using Origin 9.0.

### 2.6. Quality Assurance and Quality Control

In order to guarantee the accuracy of our results, a series of quality assurance and quality control (QA/QC) measures were taken during the process from field sampling to laboratory analysis. Three mixed soil samples were collected in each farmland. All flasks and beakers used in the experiment were washed three times with deionized water. All tests were repeated three times, and every 20 samples were used to measure one standard sample. The t-test statistical method was applied to determine whether there were significant differences among different groups. The significance level for the analysis was set at *p* < 0.05. The blank controls were produced using the same processing methods as those applied to the field samples and in laboratory process.

## 3. Results and Discussion

### 3.1. Fluorescence Characteristics of Soil DOM

The practice of 3D fluorescence spectroscopy has the advantages of requiring fewer samples, high sensitivity, and no damage to the sample structure. Compared with conventional fluorescence spectroscopy analysis, it can obtain more comprehensive DOM component information by simultaneously scanning excitation and emission wavelengths to form fluorescence excitation–emission spectral matrix (EEMS). DOM can be divided into six categories according to the different positions of fluorescence peaks. Peak A (Ex/Em = 230–260 nm/370–460 nm) represents fulvic-like substances in the ultraviolet region, which is mainly caused by some organic substances with small molecular weight and high fluorescence efficiency. The peak C (Ex/Em = 310–360 nm/370–480 nm) represents the visible light region, and it is mainly produced by organic substances with relatively stable and large molecular weight. The A and C peaks may be related to carbonyl and carboxyl groups in DOM and generally indicate exogenous inputs. Peak D (Ex/Em = 350–440 nm/430–510 nm) and F (Ex/Em = 280–288 nm/420–450 nm) represents soil humic acids, which can be used to characterize the humification degree of DOM. Peak B (Ex/Em = 225–230 nm/305–310 nm) and peak T (Ex/Em = 225–230 nm/320–350 nm) represents tyrosine-like and tryptophan-like substances, respectively, which are mainly produced by organic substances with relatively stable and large molecular weight. Fluorescence peaks B and T belong to protein-like peaks, which are usually associated with microbial decomposition [36,42,43].

**Figure 2:**
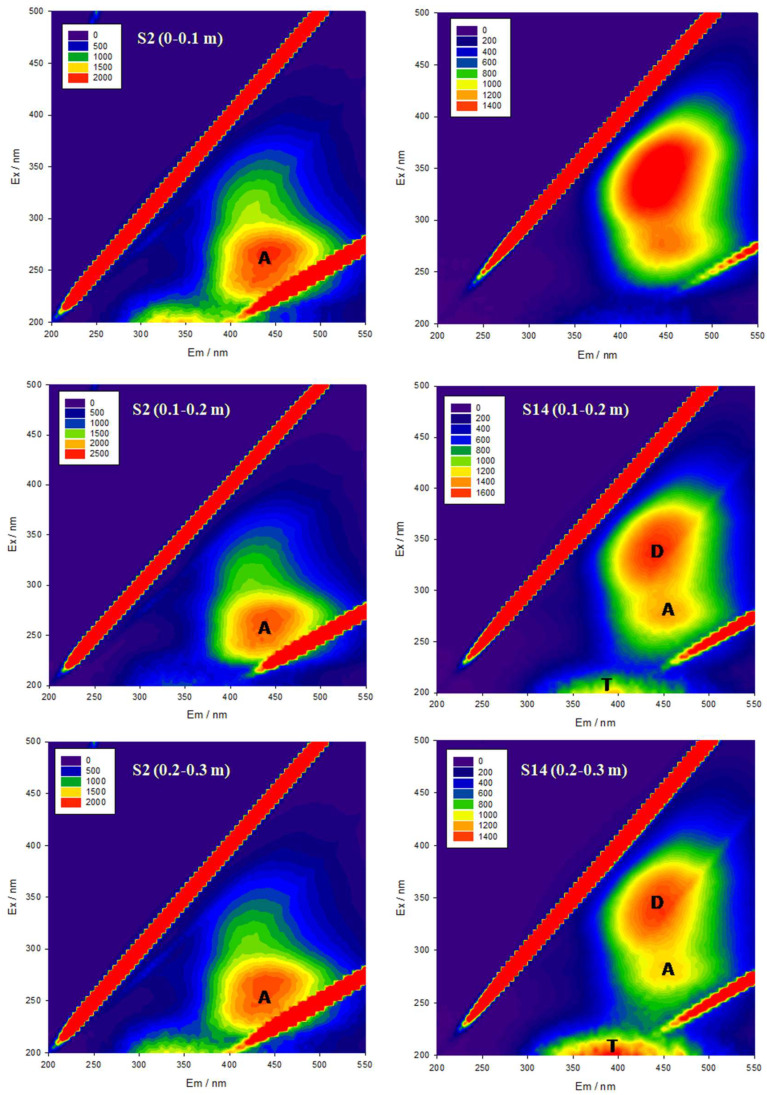
Typical 3D-EEM spectra of soil DOM of sample sitein S2 and S14.

**Figure 3:**
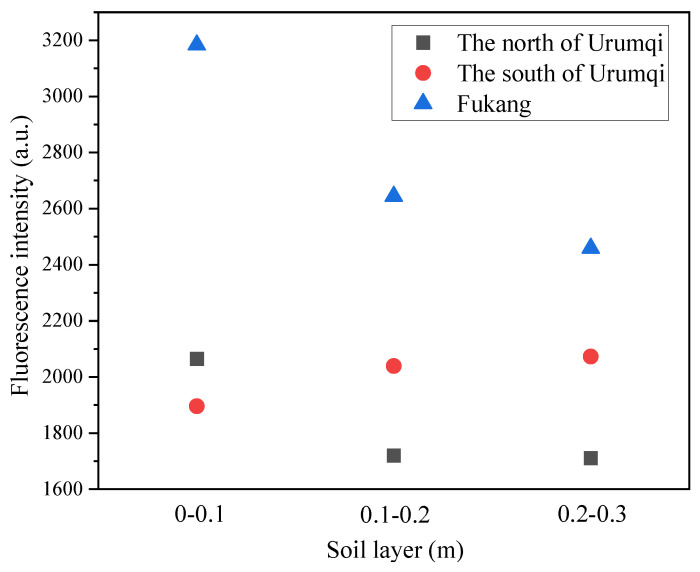
Average fluorescence intensities of different soil depth on different districts.

In this study, the fluorescence emission spectrum of the S2 sample was chosen to represent those of the north Urumqi region and Fukang, while the S14 sample was chosen to represent that of the south Urumqi region (Figure 2) according to the regional similarity features of the soil samples. The results of the fluorescence analysis showed similar peak shapes of obvious fluorescence peaks of A and D with a weak peak T in the soil DOM of the southern and northern regions of Urumqi and the Fukang region. It indicated that the DOM components of the farmland soil around Urumqi were mainly fulvic acids in the ultraviolet region, and there was lower content of soil humic acids and protein-like peak groups. The fluorescence peaks of the soil samples at different depths showed that there were only differences in fluorescence intensity without obvious difference in the peak types. The fluorescence intensity of the 0–0.1 m and 0.1–0.2 m soil samples was higher than that of the 0.2–0.3 m soil samples, probably due to the fact that the upper tilled layers of the soil contain a higher content of organic matter with higher fertility. Moreover, the root system of the crops is concentrated in 0–0.2 m layer, which may also lead to the higher fluorescence intensity in the upper layers of the soil than in the deeper layers. Further, comparing the mean fluorescence intensity in different regions (Figure 3), we found that the mean fluorescence intensity in Fukang was higher than that in the other two regions, and the mean fluorescence intensity in Fukang and the northern region of Urumqi was attenuated with soil depth, while the intensity in thesouthern part of Urumqi did not change significantly. This might be attributed to the fact that agricultural activities in the north part of Urumqi and the Fukang region are more intensive, with frequent interference, which led to more exogenous DOM in the soil, while the south part of Urumqi is mostly fallow land with much less interference, which might be the main cause of the relatively stable fluorescence intensity in this region.

**Figure 4:**
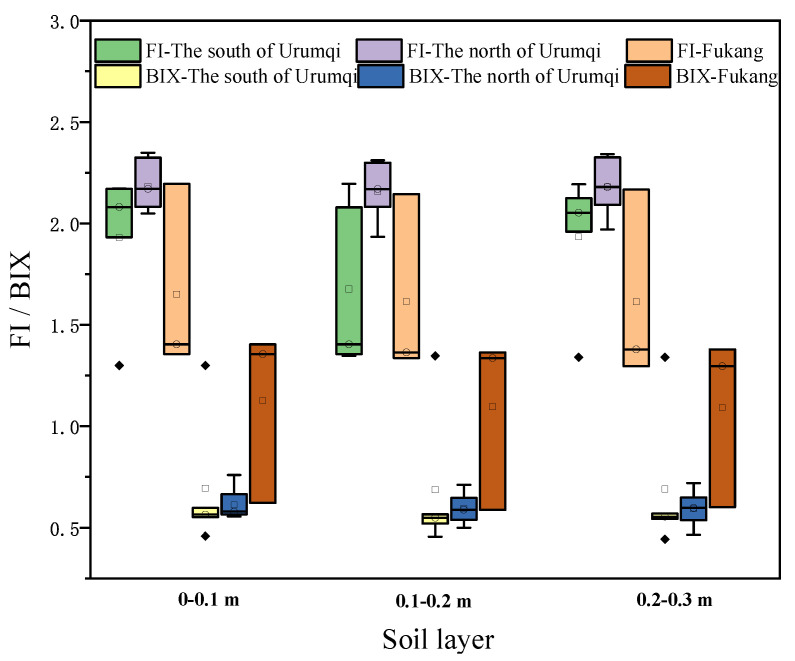
FI (fluorescence index) and BIX (autochthonous index) of soil DOM on different districts.

The fluorescence index (FI) often indicates the sources of DOM. Generally, there are two main sources of plant and microbial metabolites with FI values of 1.4 and 1.9, respectively [40,41]. When FI < 1.4, DOM is mainly from plant sources. When FI is between 1.4 and 1.9, DOM is from both plant and microbial metabolites. While FI > 1.9, DOM is dominated by microbial metabolite sources (such as tyrosine and tryptophan) [44]. In this study, as shown in Figure 4, the FI index of farmland soil DOM in the south of Urumqi and the north of Urumqi were almost higher than 1.9, indicating that DOM in these farmland soil samples mainly came from microbial metabolites [43]. The FI index of Fukang was greater than 1.4, indicating that the DOM source of Fukang farmland soil was not only microbial metabolites but also plant metabolites. There was no significant FI difference in different soil layers.

The autogenesis index (BIX) is an important index reflecting the characteristics of DOM autogenesis. When the BIX > 1, it indicates that DOM is mainly autogenic. When the BIX < 1, it indicates that DOM autogenic features are not obvious [39]. As can be seen from Figure 4, the BIX of DOM of 19 soil samples was almost less than 1, and the BIX of DOM of individual samples in Fukang was greater than 1. Therefore, it suggested that most samples did not have obvious autogenesis characteristics. They showed low bioavailability and less protein-like components, which also precisely explain why the protein-like peak was not obvious in the three-dimensional fluorescence spectrum of soil DOM.

### 3.2. UV–Vis Spectral Characteristics of Soil DOM

The nature of DOM determines the environmental effects of DOM. In addition, understanding the structural composition and main properties of DOM helps to understand the interaction between DOM and geochemical elements and pollutants. Hydrophobicity, aromatability, molecular weight, polarity, and material composition are the important properties of DOM.UV–Vis spectroscopy has the advantages of high sensitivity and lower sample requirements, and it is widely used to characterize these properties of soil DOM. Aromatability and hydrophobicity are important characterization indexes for qualitative descriptions of DOM [45]. Generally, a more aromatic and hydrophobic DOM demonstrates a stronger binding capacity with hydrophobic organic pollutants [44]. SUVA_254_ is usually used to characterize the aromaticity of DOM molecules, and higher values means greater aromaticity. As shown from Table 2, SUVA_254_ of soil DOM in our study area ranged from 0.94 to 3.07, and the aromaticity of the soil DOM was in the order of south of Urumqi > north of Urumqi > the Fukang region, with significant differences. However, the SUVA_254_ of 0–0.1 and 0.1–0.2 m soil layers was slightly higher than that of the 0.2–0.3 m soil layer, without significant differences.

The content of hydrophobic components of DOM molecules is generally defined as SUVA_260_ [46]. Higher SUVA_260_ indicates greater hydrophobic components. Table 2 shows that the range of SUVA_260_ of all samples in the study area ranged from 0.92 to 3.12. The overall variation of hydrophobicity is consistent with the aromaticity of each layer. Hydrophobic components in the 0–0.1 m and 0.1–0.2 m soil layers are slightly higher than those in the 0.2–0.3 m soil layer. This might be due to the fact that hydrophobic components are easily adsorbed to the surface soil, while hydrophilic components migrate to the deep soil. The deeper the soil, the greater the water content. In the downward migration, some components form a complex with metal ions, and the hydrogen bonds in the complex may lead to the quenching phenomenon of fluorescent substances, resulting in the stronger fluorescence intensity in the surface than in the deep layer. SUVA_260_ of different regions in Table 2 showed that SUVA_260_ in the south of Urumqi was significantly higher than that of the other two regions at each depth, followed by the north of Urumqi and the Fukang region.

Molecular weight is also an important property of DOM. Substances with small molecular weight mainly include amino acids, fatty acids, aromatic acids, etc., while substances with high molecular weight include fulvic acid and humic acid, etc. SUVA_280_ is used to characterize the molecular weight of DOM molecules, and the higher the value, the greater the molecular weight [47]. SUVA_280_ of soil samples in each layer was shown in Table 2. In comparison, the molecular weight in 0–0.1 m and 0.1–0.2 m soil was greater than that of 0.2–0.3 m, which is consistent with the features of the aforementioned sail tilled layer. Organic matter in the tilled layer was more concentrated, and the root systems were well developed. According to Table 2, the average SUVA_280_ of the samples in the south of Urumqi was significantly higher than those in the other two regions, and followed by the north of Urumqi and the Fukang region.

SUVA_250_/SUVA_365_ is used to characterize the molecular size of DOM molecules. A smaller ratio means smaller but more DOM molecules [48]. As shown in Table 2, SUVA_250_/SUVA_365_ in 0–0.2 m layer was also higher than that in the 0.2–0.3 m layer, indicating that the main DOM molecules in the surface layer were macromolecules with lower content. The deeper the soil was, the higher the DOM content and the smaller the molecules were. According to Table 2, SUVA_250_/SUVA_365_ in the south of Urumqi was significantly higher than that of the other two regions, while there was an insignificant difference in the other two regions.

SUVA_300_/SUVA_400_ is used to characterize the humification degree of DOM molecules. When SUVA_300_/SUVA_400_ > 3.5, DOM is dominated by fulic acid, and while SUVA_300_/SUVA_400_ < 3.5, humic acid is dominant. It is generally believed that the higher the degree of humification is, the stronger the binding capacity DOM has with potentially toxic elements and organic pollutants [49]. Table 2 shows that the SUVA_300_/SUVA_400_ of all soil samples was between 1.18 and 2.10 (less than 3.5), indicating that the humification degree of all soil samples was not high in three regions, and DOM was dominated by humic acid. SUVA_300_/SUVA_400_ in the south of Urumqi was higher than that in the other two regions, and the degree of soil humification in Fukang and the north of Urumqi was not significantly different. Moreover, the humification degree of the 0–0.2 m layer was slightly higher than that of the 0.2–0.3 m layer, which was similar to the trend of molecular weight and molecular size.

In general, aromaticity, hydrophobicity, molecular weight, molecular size, and humification degree of soil DOM in all three regions showed a similar trend, and these features were more obvious in the south of Urumqi than in the north of Urumqi and Fukang. The reason might be that the south of Urumqi is located in the mountainous region, and most of the land has been fallow for many years with less human disturbance, which leads to relatively rich humus in soil. Moreover, the samples in the south of Urumqi were mainly collected from greenhouse vegetables and flowers. The amount of fertilizers and other organic chemicals used in greenhouse soils was greater compared to in the field. Fertilizers could increase the content of DOM in the surface soil, reduce the C/N ratio of DOM, and improve soil fertility and conjugated structure. Fertilizers can increase humificationdegree, aromatology, hydrophobicity, and average molecular weight, which is conducive to increasing microbial activity, crop litter, and root exudates, and accelerate the decomposition and transformation of soil organic compounds [21,39]. The samples in the north of Urumqi and Fukang were mainly collected from vegetable and cotton fields, and the soil quality has been deteriorated slightly after many years of cultivation.

Compared with DOM in the 0.2–0.3 m soil layer, DOMs in the 0–0.1 m and 0.1–0.2 m soil layers are more subjective to fertilization, because the decomposition process of organic materials mainly occurs in the surface layer. In addition, long-term input of organic materials increases the microbial activity of soil in the surface layer. In addition, there is a large amount of crop residues in the surface soil, which could enrich DOM in the surface soil to a certain extent. Combined with fluorescence analysis, the surface layer was dominated by macromolecular humic acid and fulvic acid, and the humification degree was higher than that in the deep layer. However, the humification degree and the DOM molecular richness of the whole region were still very low, which was closely related to the natural environmental conditions of the relatively low soil quality of overall Xinjiang.

Therefore, we demonstrated that microbial metabolites were the dominant DOM sources around Urumqi, which was supported by FI index. Microbially-derived DOM supported less diversity of soil bacteria compared to DOM coming from plant sources. This is largely because plants represent major resource inputs to soil, and can substantially diversify the pool of resources and offer more ecological niches to partition by generating plant residues and root exudates [37,48]. In addition, we noted that all samples had no obvious autogenesis characteristics according to the BIX index. This indicates that farmland in study area is environmentally fragile and has lower microbial diversity. Meanwhile, aromaticity, hydrophobic, molecular weight, molecular size, and humification degree of soil DOM in the south of Urumqi were better than that in the north of Urumqi and Fukang, further indicating that the aromatics, hydrophobicity, humification degree, and average molecular weight of soil DOM were increased by fertilization, which made it more stable [21,39]. These results further demonstrated that DOM quality is of key importance in affecting soil quality in arid and semi-arid regions. In addition, fertilization is one of the important measures of artificial intervention and effectively enhances organic matter content in soil.

## 4. Conclusions

In this study, three-dimensional fluorescence spectroscopy and UV–visible spectrum technology were applied to characterize the structural composition and important properties of the farmland soil DOM around Urumqi city. Spectroscopic indices were used to analyze its possible sources. The results showed that the composition of DOM in the surrounding area of Urumqi was mainly humic acid and fulvic acid macromolecules, with alower content of protein-like peak group substances. The autogenetic characteristics of DOM in the three regions were not obvious. The fluorescence excitation spectra of different crop types showed a similar trend with different intensity. The fluorescence intensity of DOM in the north Urumqi and Fukang areas decreased with the increase in soil depth, while the fluorescence intensity in the south was relatively stable. The DOM content in the south of Urumqi was higher than that in the north of Urumqi and Fukang, which might be due to the use of fertilizers or organic chemicals. The higher content of DOM in upper layers of the soil (0–0.1 and 0.1–0.2 m) than in the deeper layer (0.2–0.3 m) in all three regions was due to the fact that the soil tilled layer was more subjected to fertilization and conducive to microbial activities. Spectroscopic indices analysis further confirmed that the DOM of all three regions were mainly from microbial metabolites.

## Figures and Tables

**Table 1:** Characteristics of the sampling points.

Site	Coordinates	Crop Types	Soil Types	pH	Districts
S1	87°583684′ E, 44°060206′ N	sweet potato	Camborthids	7.92	The north of Urumqi
S2	87°644409′ E, 44°082329′ N	corn	Camborthids	8.05	
S3	87°551937′ E, 44°325629′ N	cotton	Camborthids	8.32	
S4	87°595882′ E, 44°121391′ N	sweet potato	Camborthids	8.06	
S5	87°565842′ E, 44°409290′ N	cotton	Camborthids	8.39	
S6	87°591934′ E, 44°153144′ N	corn	Camborthids	8.09	
S7	87°601204′ E, 44°171739′ N	rice	Camborthids	7.93	
S8	87°631674′ E, 44°283554′ N	cotton	Camborthids	8.16	
S9	87°479110′ E, 43°976233′ N	spinach	Camborthids	7.96	
S10	87°472458′ E, 43°963415′ N	wheat	Camborthids	8.07	
S11	87°584381′ E, 44°229457′ N	pumpkin	Camborthids	8.07	
S12	87°402805′ E, 43°459628′ N	greenhouse flower	Calciustoll	7.56	The south of Urumqi
S13	87°282321′ E, 43°480383′ N	greenhouse flower	Calciustoll	7.68	
S14	87°390822′ E, 43°629482′ N	greenhouse vegetable	Calciustoll	7.93	
S15	87°355654′ E, 43°597425′ N	potato	Calciustoll	8.03	
S16	87°389653′ E, 43°682755′ N	onion	Calciustoll	8.05	
S17	87°961693′ E, 44°339934′ N	cotton	Quartisamment	8.36	Fukang
S18	88°474617′ E, 44°166691′ N	corn	Quartisamment	8.40	
S19	88°303642’ E, 44°172170′ N	corn	Quartisamment	8.51	

**Table 2:** The characteristics of UV spectra absorption of soil DOM on different districts (SUVA_254_, SUVA_260_, SUVA_280_, SUVA_250_/SUVA_365_, SUVA_300_/SUVA_400_).

Site	SUVA_254_/L·(mg·m)^−1^			SUVA_260_/L·(mg·m)^−1^			SUVA_280_/L·(mg·m)^−1^			SUVA_250_/SUVA_365_			SUVA_300_/SUVA_400_			District
	0–0.1 m	0.1–0.2 m	0.2–0.3 m	0–0.1 m	0.1–0.2 m	0.2–0.3 m	0–0.1 m	0.1–0.2 m	0.2–0.3 m	0–0.1 m	0.1–0.2 m	0.2–0.3 m	0–0.1 m	0.1–0.2 m	0.2–0.3 m	
S1	1.4552	1.4911	1.4606	1.5753	1.5780	1.5114	1.4588	1.4670	1.3927	1.4609	1.6430	1.5954	1.2820	1.3833	1.3666	The north of Urumqi
S2	1.1731	1.1270	1.1138	1.2464	1.2139	1.1967	1.1958	1.1662	1.1610	1.4235	1.4347	1.3925	1.2936	1.2852	1.2567	
S3	1.4295	1.2909	1.2082	1.3753	1.2980	1.2263	1.3860	1.2788	1.2324	1.5678	1.5179	1.4679	1.3196	1.3066	1.2775	
S4	1.1023	1.1323	0.9488	1.0823	0.9674	0.9235	1.0635	1.0132	0.9721	1.5577	1.3623	1.3049	1.2555	1.2101	1.1836	
S5	0.9893	0.9753	0.9461	1.0098	1.0110	0.9648	1.0353	1.0350	1.0025	1.3858	1.3949	1.3587	1.2350	1.2396	1.2360	
S6	1.3878	1.3381	1.8280	1.3480	1.3033	1.8365	1.2978	1.2704	1.6815	1.6349	1.5974	1.8640	1.3763	1.3687	1.5065	
S7	0.9802	1.1823	1.0801	1.0377	1.2432	1.1394	1.0416	1.1766	1.1021	1.3678	1.4510	1.3806	1.2350	1.2499	1.2411	
S8	1.1600	1.4786	1.2913	1.1384	1.4330	1.2708	1.0994	1.1636	1.0996	1.4710	1.5919	1.4929	1.2952	1.3900	1.3024	
S9	1.4158	1.6456	1.5728	1.3643	1.5845	1.5238	1.2070	1.2969	1.2930	1.6525	1.6454	1.6624	1.3099	1.4169	1.4251	
S10	1.2560	1.2279	1.0795	1.2065	1.1683	1.0329	1.0810	1.1049	0.9731	1.4962	1.4677	1.4237	1.3386	1.2971	1.2941	
S11	1.1979	1.3143	1.5668	1.1395	1.2276	1.4040	1.0366	1.1269	1.2792	1.4510	1.5078	1.5893	1.2900	1.3405	1.3882	
S12	2.8835	2.8842	3.0710	2.8904	2.9074	3.1274	2.5993	2.5649	2.7767	2.4165	2.4034	2.4553	2.1037	2.0073	2.0381	The south of Urumqi
S13	2.5336	2.1703	2.2660	2.4406	2.1124	2.1979	2.2232	1.9014	2.0591	2.2873	2.1623	2.1273	1.9308	1.7553	1.6863	
S14	1.1083	2.3971	1.8065	1.2152	2.4163	1.8783	1.1870	2.1330	1.6689	1.3554	1.9819	1.6741	1.2512	1.5987	1.4626	
S15	1.4164	1.8039	1.1916	1.4721	1.8453	1.2287	1.3794	1.6426	1.1785	1.5443	1.8303	1.4504	1.3403	1.5631	1.3092	
S16	1.2025	1.3446	1.2895	1.1420	1.1200	1.2078	1.0487	1.1530	1.0950	1.4835	1.5565	1.5333	1.2994	1.3472	1.3399	
S17	1.1342	1.0790	1.0905	1.1312	1.1257	1.1309	1.1382	1.0984	1.1283	1.4898	1.4282	1.4335	1.3194	1.2644	1.2813	Fukang
S18	1.2572	1.2672	1.3409	1.2240	1.2357	1.3033	1.1185	1.1196	1.1892	1.5377	1.5496	1.5326	1.3556	1.3645	1.3788	
S19	1.3434	1.2495	1.2023	1.1731	1.1675	1.1319	1.1297	1.0440	1.0579	1.6366	1.5378	1.4780	1.4037	1.3363	1.2958	

## Data Availability

This study does not report any data.
